# An Insight into Immunological Therapeutic Approach against Cancer: Potential Anti-cancer Vaccines

**DOI:** 10.2174/0113892029319505240821063238

**Published:** 2024-08-30

**Authors:** Arjun Singh Kohli, Somali Sanyal, Radhey Shyam Kaushal, Manish Dwivedi

**Affiliations:** 1 Amity Institute of Biotechnology, Amity University Uttar Pradesh, Lucknow Campus, Gomtinagar Ext., Lucknow, 226028, India;; 2 Department of Biotechnology, Parul University, Gujrat, India;; 3 Research Cell, Amity University Uttar Pradesh, Lucknow Campus, India

**Keywords:** Cancer, vaccine, immunotherapy, therapeutics, precision therapy, cancerous cells

## Abstract

The development of a cancer vaccine comes with its complications and designing and developing a vaccine against foreign invaders such as bacterial and viral particles is not as complex and multi-faceted as the preparation of immunotherapy for host-infected cells which resemble our own body cells. The entire research and development framework of designing a vaccine for cancerous cells lies entirely on the remarkable aspect of notifying specific interactions and acclimatising the immune system. This review aims to compile the several fronts research-based methodology applies to in terms of developing a therapeutic, preventive or personalised vaccine for cancer. The approach lays focus on the identification and selection of targets for vaccine development which have come to light as immune biomarkers. Furthemore, significant aspects of personalised and precision vaccines and the fine line that runs between these approaches have also been discussed.

## 1 INTRODUCTION

The practice of immunising against infectious agents to avoid illness by developing humoral immunity is where the concept of the vaccine originates. People receive vaccinations against viral or bacterial antigens before coming into contact with pathogenic organisms. Because viral genes are generally straightforward and only include a small number of specified antigens, this method became quickly effective for viruses. However, there are an infinite number of possible antigens that may be the focus of an immune response in the case of the majority of cancer [1].

Cancer continues to pose immense challenges in terms of treatment and cure, impacting millions of individuals worldwide. In recent years, there has been a surge of interest and optimism among researchers and medical professionals in exploring innovative strategies to combat this intricate and diverse disease [2]. One such strategy that has garnered substantial attention and hope is the development of cancer vaccines which belong to the realm of immunotherapy, harnessing the immune system's capabilities to recognise and eliminate cancer cells. Unlike conventional preventive vaccines that primarily focus on preventing infectious diseases, cancer vaccines are designed to trigger and enhance the body's natural immune response against tumour cells. They function by stimulating the immune system to identify unique antigens, proteins, or markers specific to cancer cells, thereby initiating a targeted immune attack to eradicate the tumour. Cancer vaccines offer a promising avenue in the field of oncology, holding the potential for a significant breakthrough in both preventing and treating various types of cancer [3]. They represent an innovative approach to the battle against cancer. By harnessing and augmenting the immune system's response to tumour cells, they hold tremendous potential for preventing, treating, and potentially eradicating various types of cancer.^10^Traditional cancer treatments, such as surgery, chemotherapy, and radiation therapy, although made remarkable progress in improving patient outcomes often exhibit limitations such as adverse side effects and the inability to effectively target metastatic or recurrent tumours. Consequently, there is an urgent need for innovative therapies that can provide targeted, specific, and long-lasting responses against cancer cells while minimizing harm to healthy tissues [4]. Cancer vaccines offer several advantages over conventional cancer treatments. They can be highly targeted, focusing on specific tumour-associated antigens, thereby minimizing damage to healthy cells and reducing side effects. Cancer vaccines also hold promise in stimulating immune responses against both primary tumours and metastatic lesions, making them a viable option for advanced or widespread cancers [5-11]. Furthermore, unlike traditional chemotherapy or radiation therapy, cancer vaccines can establish a durable immune memory, enabling the immune system to recognize and eliminate recurrent tumour cells, should they re-emerge [12].

Currently, numerous types of cancer vaccines are under development and investigation. One approach involves the use of preventive vaccines to guard against the development of certain cancers associated with viral infections, such as the human papillomavirus (HPV) and hepatitis B virus (HBV) [6, 7]. Vaccines like the HPV vaccine, for instance, have the potential to reduce the risk of developing HPV-related cancers like cervical, anal, and oropharyngeal cancers [5]. Another approach involves therapeutic vaccines, which aim to treat existing cancers by boosting the immune response against tumour cells. Therapeutic cancer vaccines can be broadly classified into two main types: peptide/protein-based vaccines and dendritic cell-based vaccines. Peptide or protein-based vaccines incorporate specific tumour antigens, either synthetic or derived from the patient's tumour cells, to stimulate an immune response [6]. Dendritic cell-based vaccines, on the other hand, involve isolating a patient's dendritic cells (specialized immune cells), loading them with tumour antigens, and reintroducing them into the patient's body to provoke a robust immune response [8]. Cancer vaccines employ diverse mechanisms to induce anti-tumour immune responses. They can enhance the activity of immune cells such as cytotoxic T-cells and natural killer cells, which directly identify and eliminate cancer cells. Additionally, vaccines can stimulate the production of specific antibodies that target cancer cells or disrupt tumour-promoting signals within the body [9]. By leveraging the immune system's remarkable specificity and memory, cancer vaccines have the potential to provide enduring protection against cancer recurrence and metastasis, ultimately leading to improved patient outcomes [8, 10].

Age and gender are significant considerable parameters while administering vaccines to population pools and the efficacy of any vaccine type may vary per the recipient’s age and gender. In case of cancer vaccines, similar considerations are necessary before vaccinations. A study with patients with stage IIB-IV resected melanoma were recruited for three clinical studies in which they were immunised six times with twelve different adjuvants including Class I MHC restricted peptides from melanocytic differentiation antigens and cancer testis antigens. The IFN-gamma ELIspot test was used to directly detect T-cell responses. Age and gender were among the clinical data that were gathered. For every variable, the cumulative incidence of the immunological response across time was plotted. A discernible immune response deterioration with advancing age was reported, however, a significant percentage of the elderly showed immunity to vaccinations. Hence, elderly patients must not be excluded from any cancer vaccination schedules. On the other hand, despite potent evidence implying females developing immunoprotection, gender has been seen to not affect the efficacy of cancer vaccines.

To date, different studies have reported the types or the development strategies of cancer vaccines. However, the information is scattered. The present review will provide collective details on different types of cancer vaccines and their developmental strategies which will give clear and comprehensive ideas about cancer vaccine and their potential as an immunological therapeutic approach against cancer.

## 2 APPROACHES ADOPTED FOR SELECTION OF ANTIGENS FOR THE DEVELOPMENT OF CANCER VACCINES

The careful selection of antigens is a crucial step in the development of cancer vaccines, as it determines their effectiveness in triggering targeted immune responses against cancer cells. A variety of approaches have been adopted to identify and choose antigens that can induce potent and specific immune responses [13-17]. These approaches include targeting tumour-specific antigens (TSAs), tumour-associated antigens (TAAs), viral antigens, and employing combinatorial strategies. Each of these approaches plays a vital role in designing successful cancer vaccines [14].

### 2.1 Tumour-specific Antigens (TSA) as Target

TSAs are unique to cancer cells and arise from genetic alterations such as mutations, gene fusions, or viral integration [16]. To identify TSAs, advanced sequencing techniques like whole-exome sequencing (WES) and whole-genome sequencing (WGS) are commonly utilized. These techniques enable researchers to identify genetic alterations specific to cancer cells by analysing the sequencing data. Where WES involves deep sequencing of sample tumour exome for the assessment of somatic mutations, WGS, on the other hand, requires genomic sequencing of the sample to establish comparisons between the tumour and normal tissue DNA for specific gene products such as Single Nucleotide Variants (SNVs) and Insertions or Deletions (INDELs). Among TSAs, neoantigens hold great promise [18]. Neoantigens are derived from somatic mutations and are highly specific to an individual patient's tumour. Computational algorithms can predict neoantigens based on tumour sequencing data, facilitating the development of personalized cancer vaccines [19]. Cancer-testis antigens (CTAs) are another type of TSA that are typically expressed in the testis but are also found in various cancer types. CTAs are attractive targets due to their tumour-specific expression and immunogenicity [20].

### 2.2 Tumour-associated Antigens (TAA) as Target

TAAs are expressed in both cancer cells and normal cells but are often overexpressed or aberrantly expressed in cancer, making them potential targets for cancer vaccines. Techniques like gene expression profiling, proteomics, and comparative analysis of cancer and normal tissues are used to identify TAAs [21]. Gene expression profiling allows researchers to identify genes that are either upregulated or downregulated in cancer cells compared to normal cells. Proteomic approaches involve analysing the patterns of protein expression in cancer cells and normal cells to identify differentially expressed proteins. Comparative analysis of cancer and normal tissues enables the identification of antigens that are selectively expressed or overexpressed in cancer cells. TAAs are poor immunogens since they are self-antigens, hence, any T-cells generated specific to TAAs are deleted primarily. To overcome this drawback, antigen modification is essential which induces cross-reactivity *via* epitope modifications. Self TAA from one specific species can cross-react with a foreign TAA from another species thus improving the overall immunogencity of the antigens. Examples of TAAs include HER2/neu, carcinoembryonic antigen (CEA), melanoma-associated antigen (MAGE), and prostate-specific antigen (PSA) [22] and most of the vaccines based on these antigens have completed the trial phase -III (Table **1**).

### 2.3 Shared Antigens as Target

Shared antigens are expressed in both cancer cells and normal cells but are overexpressed or aberrantly expressed in cancer, which makes them potential targets for immune recognition [23]. These antigens are often identified through comparative analysis of gene expression profiles between cancer and normal tissues or through proteomic approaches. Commonly found in patient subgroups of tumour types, shared antigens are those that are expressed in a high enough percentage of patients to allow vaccine developers to target these patient groups with routine testing. By comparing the expression levels of genes or proteins in cancer cells and normal cells, researchers can identify antigens that are more abundant in cancer cells [24]. Thus, both TSAs and TAAs can be targeted by shared antigen vaccines. The TSA human papillomavirus E6 and E7 proteins (HPV E6 and E7) are expressed in approximately 60% of oropharyngeal cancers and nearly all cervical cancers. The TAA Wilms' tumour protein (WT1) is overexpressed in the majority of acute myeloid leukaemias (AMLs), breast cancers, and glioblastomas (GBMs). For example, the neo-epitope TSA epidermal growth factor receptor variant III (EGFRvIII) is expressed in approximately 25% of EGFR-overexpressing glioblastomas (GBMs). Personalised antigen vaccines are not the same as shared antigen vaccines because the former can be evaluated using common tests like immunohistochemistry, flow cytometry, and cytology. Since the 1990s, predefined, shared antigen vaccines have dominated preclinical and clinical research and offered fundamental insights. Shared antigens may also include oncofoetal antigens like alpha-fetoprotein (AFP), which are expressed during foetal development but are re-expressed in certain cancers [25].

### 2.4 Viral Antigens Expressed on Cancer Cells as Target

Some cancers are associated with viral infections, and viral antigens expressed in tumour cells can be targeted for cancer vaccines. Techniques such as viral genome sequencing, proteomic analysis, or detection of virus-specific antibodies are used to identify viral antigens. Viral genome sequencing helps researchers identify viral proteins expressed in infected tumour cells. Proteomic analysis involves identifying and characterising viral proteins using mass spectrometry-based approaches. Additionally, the detection of virus-specific antibodies in patient sera can indicate the presence of viral antigens in cancer cells [26, 27].

Globally, hepatocellular carcinoma (HCC) is among the most common and deadly illnesses. It is shown that the development of HCC is directly correlated with hepatitis virus type B (HBV) or type C (HCV) infection. The double-strand DNA virus known as HBV has four overlapping reading frames and a genome of about 3.2 kbp. The three main ways that HBV causes carcinogenesis are as follows: Three main processes are mediated by HBV: (1) integration of HBV DNA into the host genome; (2) cellular signing route; and (3) virus-induced chronic inflammation [25]. HBV-related oncoproteins are produced and involved in carcinogenesis in the first two pathways; these oncoproteins may serve as targets for immune cell identification. The X gene encodes the most well-known oncoprotein, HBV X (HBx), which is involved in signalling pathways such as MAPK and JNK to increase proliferation and interfere with DNA repair mechanisms, ultimately leading to a malignant phenotype. Numerous vaccines against HBV-related HCC were investigated for their therapeutic efficacy against HCC *in vivo* and *in vitro*, based on the HBx oncoprotein [25, 26].

### 2.5 Dendritic Cells-based Approaches

**Fig. (1):**
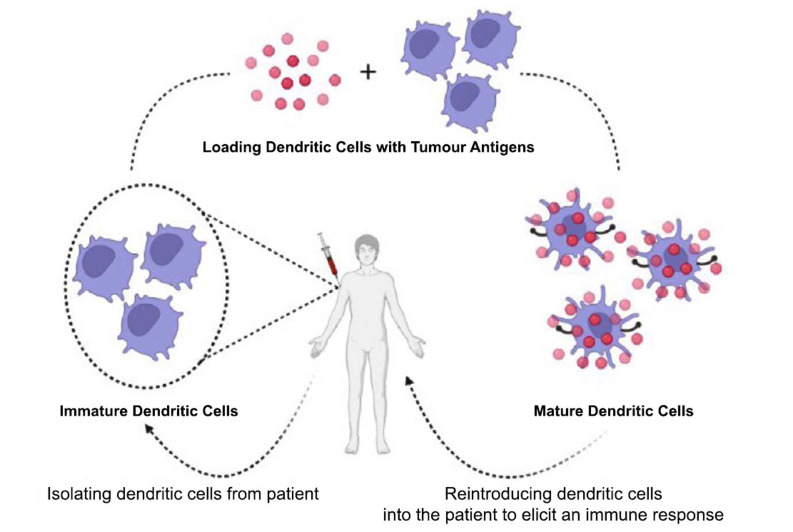
Dendritic cells-based approaches for the development of cancer vaccines. Steps involved in dendritic cells-based approaches: Isolation of CD14+ monocytes/ CD34+ hematopoietic cells/ natural dendritic cells from an oncological patient *via* apheresis; Addition of dendritic cell differentiation factors (CD14+ monocytes/ CD34+ hematopoietic cells) to obtain immature dendritic cells; Addition of cytokine cocktail for dendritic cell maturation or electroporation with CD40L, CD70, _CA_TLR4 mRNA; Introduction of the prepared formulation into the potential recipient *via* intratumoural/ intravenous/ intranodal/ intradermal pathway; On introduction, the mature dendritic cells migrate to the lymph nodes, where antigen presentation to the naïve CD8+ T-cells, CD4+ T-cells and B-cells takes place and then the respective activated cells migrate to the tumour.

Dendritic cell-based approaches utilize the powerful antigen-presenting cells called dendritic cells (DCs) to initiate and regulate immune responses. These approaches involve isolating DCs from a patient, loading them with tumour antigens, and reintroducing them into the patient to stimulate an immune response [28]. Tumour antigens used in DC-based approaches can be obtained from various sources, including tumour lysates, tumour mRNA, or synthetic peptides [29]. Tumour lysates contain a mixture of antigens derived from whole tumour cells, including both TSAs and TAAs. Tumour mRNA can be isolated from cancer cells and used to produce specific tumour antigens in the form of RNA-loaded DCs. Synthetic peptides representing specific tumour antigens can also be utilized (Fig. **1**) [30].

### 2.6 Mixture of Tumour Cells or Lysates as the Antigen Source

Whole tumour cell vaccines employ a mixture of tumour cells or lysates as the antigen source. These vaccines contain a wide range of tumour-associated antigens, including both TSAs and TAAs [29, 31]. Autologous tumour cells can be obtained through biopsies or surgical resection, and these cells are processed to generate tumour cell lysates, which are then used as the antigen source for the vaccine. Strategies for autologous cancer vaccinations create customised treatments using tissue taken from the patient's tumour. These therapies range widely, from using irradiated cancer cells that were obtained from the patient to induce anti-tumour immunity to vaccination with patient-specific purified cancer antigens. It is possible to further customise cell-based treatments by transfecting them with extra immune stimulatory molecules. To directly stimulate the immune system, these autologous immunotherapies comprise a broad variety of proteins and peptides specific to the individual's tumour antigens presented by self-major histocompatibility complex (MHC). Furthermore, current research has shown that a very diverse population of cancer cells make up tumours. Thus, it is possible that a whole primary tumour mass may be needed for an effective vaccination, and that autologous cell lines may not be adequate to elicit a broad immune response [32].

Except that the material used in allogeneic vaccines comes from a different member of the same species, they are essentially the same as autologous vaccinations. Allogeneic materials are frequently employed, such as proven cancer cell lines cultured in laboratories that are known to express TAAs unique to a particular tumour type. This makes it possible to generate, store, and alter these medicines in large quantities before using them. Allogeneic cell-based vaccines may differ from autologous vaccines in that they do not contain “patient-specific” tumour antigens, despite the fact that many of them are derived from cancer cell lines of the same species and kind (breast, prostate, lung, *etc*.) [31]. Allogeneic derived vaccines have an additional benefit, however, as preclinical research in models of melanoma and prostate cancer have shown that presenting TAAs in an allogeneic context boosts immunogenicity. Mismatched minor histocompatibility antigen bone marrow transplant recipients exhibit higher graft *vs*. tumour responses, which supports the theory that allogeneic cells offer an extra warning signal [32].

Furthermore, allogeneic immunotherapy is a noteworthy method due to its availability, low cost of manufacture, and lack of invasive procedures, among other desired attributes [32].

The HyperAcute^®^ pancreatic cancer vaccine (Algenpantucel-L) from NewLink Genetics and the GVAX pancreas vaccine from BioSante Pharmaceuticals are two allogeneic vaccinations that are presently being investigated in clinical studies for the treatment of pancreatic cancer. Combining two human allogeneic pancreatic cancer cell lines that express α-1,3-galactosyltransferase, a murine enzyme that promotes α-galactosyl (αGal) epitope expression on the cell surface, results in Algenpantucel-L (HyperAcute-Pancreas). The idea behind this vaccination approach is to use the adjuvant effect of naturally occurring anti-αGal antibodies produced by the human body. the immunological reaction against the tumour that is brought about by complement activation, opsonization, and Antibody-dependent-cellular Cytotoxicity (ADCC) of anti-αGal immune complexes [32].

### 2.7 Combinatorial Approaches

Combinatorial approaches involve the use of multiple antigens in cancer vaccines to target various aspects of tumour biology and enhance the immune response. These approaches may include the inclusion of TSAs, TAAs, viral antigens, or other tumour-specific markers [33]. The rationale behind combinatorial approaches is to broaden the immune response, reduce the chance of immune escape by tumour cells, and increase the likelihood of clinical efficacy. By targeting multiple antigens, the vaccine aims to activate different immune cell populations, such as CD8+ T-cells, CD4+ T-cells, and B cells, resulting in a more robust and comprehensive immune response against cancer cells [34-38].

The selection of antigens for cancer vaccines involves a thorough analysis of tumour-specific, tumour-associated, or shared antigens [35, 39]. Various approaches, including genomic profiling, proteomics, and immunological assays, are employed to identify and validate candidate antigens. The choice of antigens depends on their expression patterns, immunogenicity, and potential for therapeutic targeting. By leveraging these approaches, researchers strive to develop cancer vaccines that can effectively stimulate the immune system to recognize and eliminate cancer cells, ultimately leading to improved patient outcomes [40, 41].

Many whole cell-based vaccinations such as tumour-infiltrating lymphocytes (TIL), T-cell receptor (TCR), or chimeric antigen receptor (CAR)-modified T-cells, as well as dendritic cell (DC)-based vaccines are now being studied in a variety of clinical trials utilising two or more combinations. The co-administration of PD-L1 siRNA with a DC-based mRNA vaccination is an intriguing new strategy that boosted anti-tumour responses by downregulating PD-L1 in tumour-antigen-presenting DCs [40]. The main obstacles to combining whole cell-based vaccinations with immune checkpoint inhibitors (iCPIs) are adverse outcomes brought on by toxicities and autoimmunity, which need to be minimised even if early research produced encouraging results [39, 40].

## 3 VARIATIONS IN CANCER VACCINES

Cancer vaccines can be grouped into several categories based on their mechanism of action, target antigens, and the type of immune response they elicit resulting in prevention as well as treatment of cancer.

### 3.1 Preventive Cancer Vaccines

Preventive cancer vaccines have emerged as a potent tool in the battle against cancer, with the primary objective of reducing the occurrence of specific types of cancer by targeting viruses that are known to be linked to cancer development [39]. These vaccines have shown remarkable success in preventing viral infections, consequently reducing the risk of developing associated cancers [40]. Viruses play a significant role in the development of certain cancers, and the targeting of these viral infections can disrupt the chain of events that lead to malignancy [39, 41]. One notable example of a preventive cancer vaccine is the human papillomavirus (HPV) vaccine. HPV is a sexually transmitted infection that can cause various types of cancer, such as cervical and anal cancer. The HPV vaccine has proven highly effective in preventing HPV infections and reducing the risk of HPV-associated cancers [41].

A study published in The Lancet in 2020 analysed data from 60 million individuals and found that the prevalence of HPV infections decreased by 86% among teenage girls and by 71% among women in their early 20s in countries with high vaccination coverage. This study highlights the substantial impact of the HPV vaccine in preventing viral infections that can lead to cervical cancer and other HPV-related malignancies [42]. Another notable preventive cancer vaccine targets the hepatitis B virus (HBV), a major risk factor for liver cancer. The hepatitis B vaccine has played a crucial role in preventing HBV infections and subsequent cases of liver cancer. In 2019, over 84% of infants globally received three doses of the hepatitis B vaccine, according to the World Health Organization (WHO). This widespread vaccination has led to a significant decline in the prevalence of HBV infection, particularly in regions where the vaccine has been integrated into national immunization programs [43]. Major FDA-approved vaccines against HPV and HBV are mentioned in Table **2**.

The impact of preventive cancer vaccines goes beyond HPV and HBV. Researchers are actively investigating other viral risk factors and developing vaccines to target them. For instance, the development of a vaccine against the Epstein-Barr virus (EBV) is underway. EBV is associated with several malignancies, including Burkitt lymphoma, Hodgkin lymphoma, and nasopharyngeal carcinoma [42, 44]. Preliminary studies have shown promising results in the development of an EBV vaccine, with the potential to reduce the incidence of these cancers in the future [45]. While viral infections receive significant attention in the realm of preventive cancer vaccines, non-viral risk factors are also being explored. For example, researchers are investigating the development of a vaccine targeting high-risk strains of the bacteria Helicobacter pylori (H. pylori). H. pylori infection is a well-established risk factor for stomach cancer. A preventive vaccine targeting H. pylori could potentially provide long-term protection against this type of cancer [46].

The development of preventive cancer vaccines involves rigorous research, clinical trials, and regulatory approvals. Preclinical studies aim to identify viral or bacterial antigens that elicit robust immune responses and play a role in cancer development. These antigens are then formulated into vaccines, often combined with adjuvants that enhance the immune response [47].

Clinical trials are crucial in evaluating the safety, efficacy, and optimal dosage of preventive cancer vaccines. These trials involve large populations and can span several years to gather comprehensive data on vaccine performance [48, 49]. Through these trials, vaccines are thoroughly tested to determine their ability to prevent viral infections and subsequent cancer development.

Combination therapies also hold promise in enhancing the efficacy of preventive cancer vaccines. Researchers are exploring the use of checkpoint inhibitors, immune-stimulating agents, or other immunotherapies in conjunction with vaccines to augment the immune response and improve overall patient outcomes. These combinations aim to overcome immunosuppressive mechanisms employed by cancer cells, allowing the immune system to better recognize and eliminate cancer cells [44, 45].

Despite the significant success of preventive cancer vaccines, challenges persist in achieving high vaccination coverage rates globally. Limited access to vaccines, vaccine hesitancy, and socioeconomic disparities can hinder the full potential of these preventive strategies. Addressing these challenges requires collaborative efforts between governments, healthcare providers, researchers, and community organizations to promote vaccine education, accessibility, and acceptance [47]. Public health initiatives are instrumental in increasing awareness and improving vaccination rates. Incorporating preventive cancer vaccines into routine immunization schedules has shown promising results. For example, Australia implemented a national HPV vaccination program in 2007, targeting both males and females. As a result, Australia has witnessed a substantial decline in the prevalence of HPV infections and related diseases. This successful program serves as a model for other countries seeking to maximize the impact of preventive cancer vaccines. It is important to acknowledge that preventive cancer vaccines are not universally effective against all types of cancer. They primarily target specific viral or bacterial risk factors that are associated with malignancies. Therefore, ongoing research aims to identify additional risk factors, including genetic and environmental factors, to expand the range of preventable cancers and develop new preventive strategies [46, 47, 50-52].

### 3.2 Therapeutic Cancer Vaccines

While traditional cancer treatments like surgery, chemotherapy, and radiation therapy have made significant progress, the need for more effective and targeted therapies is growing. Therapeutic cancer vaccines have emerged as a promising approach to stimulate the body's immune system in recognizing and eliminating cancer cells [53-55]. Recent advancements and ongoing research have contributed to the development of therapeutic cancer vaccines (Table ** 3**).

Therapeutic cancer vaccines are a type of immunotherapy designed to treat existing cancers by harnessing the immune system [56]. Unlike preventive vaccines that aim to prevent infectious diseases, therapeutic vaccines assist the immune system in combating established cancer [57]. These vaccines introduce specific cancer antigens or immune-stimulating molecules into the body, triggering an immune response against cancer cells [57]. The immune response elicited by therapeutic cancer vaccines involves both the innate and adaptive immune systems. The innate immune system recognizes danger signals released by cancer cells, while the adaptive immune system mounts a targeted response against cancer-specific antigens [58-62]. This immune activation can lead to the destruction of cancer cells and the development of immunological memory to prevent cancer recurrence [47].

Recent advancements in genomic sequencing and understanding tumour heterogeneity have paved the way for personalized cancer vaccines. By analysing the genetic profile of a patient's tumour, scientists can identify specific antigens unique to the patient's cancer cells [63]. This information enables the development of personalized vaccines tailored to each patient's specific cancer, thereby enhancing treatment efficacy [63]. A notable example of personalized cancer vaccines is neoantigen-based vaccines. Neoantigens are mutated proteins found exclusively in cancer cells and not in healthy tissues [64]. By identifying neoantigens, researchers can design vaccines that specifically target these mutated proteins, eliciting a potent immune response against the cancer cells harbouring them [65].

Combining therapeutic cancer vaccines with other immunotherapies has shown significant promise in enhancing treatment outcomes [66]. Immune checkpoint inhibitors, for instance, release the brakes on the immune system, allowing it to mount a more robust response against cancer cells. When combined with therapeutic vaccines, checkpoint inhibitors amplify the immune system's ability to recognize and attack cancer cells [66-68].

**Fig. (2):**
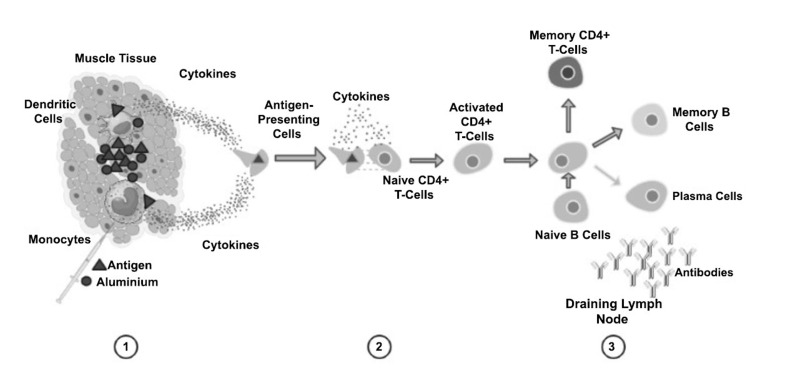
Mode of action of Aluminium adjuvants in cancer treatment. Step (**1**): The prepared formulation is introduced into the patient muscle. Aluminium activates monocytes and dendritic cells and impacts antigen uptake. Step (**2**): Monocytes and dendritic cells, once activated, present the antigen to CD4+ T-cells in the lymph node. Cytokine signalling and co-stimulatory mechanisms are triggered to stimulate T- cells. Step (**3**): B-cell response mechanisms are activated with the support of activated CD4+ T-cells.

Recent clinical trials have demonstrated the efficacy of combination therapies. In 2018, the FDA approved the combination of nivolumab and ipilimumab, two immune checkpoint inhibitors, for the treatment of metastatic melanoma. This milestone approval showcased the potential of combining immunotherapies and therapeutic vaccines to achieve improved patient outcomes [69]. Adjuvants are substances that enhance the immune response triggered by vaccines. Recent advancements in adjuvant development have played a critical role in improving the efficacy of therapeutic cancer vaccines. Adjuvants such as toll-like receptor agonists and cytokines help amplify and direct the immune response, making the vaccines more potent. Additionally, the development of novel delivery systems has contributed to the progress of therapeutic cancer vaccines. Nanoparticle-based delivery systems, for example, can encapsulate antigens and adjuvants, allowing for controlled release and targeted delivery to immune cells. This approach enhances the stability of vaccines, improves antigen presentation, and enhances immune response activation (Fig. **2**) [66].

In recent years, clinical trials have focused on a range of cancer types, including melanoma, lung, breast, and colorectal cancers. For instance, a phase III trial investigating the MAGE-A3 vaccine in non-small cell lung cancer did not meet its primary endpoint. However, further analysis revealed a potential benefit in a subset of patients with a specific genetic profile, highlighting the importance of personalized approaches in therapeutic cancer vaccine development [62-64, 67]. Despite the progress, several challenges must be addressed. The complexity of the immune system and the heterogeneity of cancer cells pose significant hurdles. Identifying optimal antigen targets that are specific to cancer cells while sparing healthy tissues is crucial. Moreover, enhancing vaccine efficacy, overcoming immune tolerance mechanisms, and determining the most effective timing and combination of therapies require further research [68]. The field of therapeutic cancer vaccines is rapidly evolving, with several exciting developments on the horizon. Here are some future directions and emerging technologies that hold promise:

#### 3.2.1 RNA-based Vaccines

RNA-based vaccines, such as mRNA vaccines, have gained significant attention following their success in COVID-19 vaccine development. These vaccines can be rapidly synthesized and offer the advantage of easily encoding multiple antigen targets. RNA-based therapeutic cancer vaccines are being explored in clinical trials, showing the potential to stimulate immune responses against cancer-specific antigens [69].

#### 3.2.2 Oncolytic Viruses

Oncolytic viruses are genetically modified viruses that selectively infect and replicate within cancer cells, leading to their destruction. These viruses not only directly kill cancer cells but also trigger an immune response against the tumour. Combining oncolytic viruses with therapeutic cancer vaccines has the potential to enhance the immune response and improve treatment outcomes [65, 69].

#### 3.2.3 Tumour Microenvironment Modulator

The tumour microenvironment plays a crucial role in cancer progression and immune evasion. Modulating the tumour microenvironment to make it more conducive to immune cell infiltration and activation is a promising approach [70, 71]. Combining therapeutic vaccines with agents that target the tumour microenvironment, such as immune-modulating drugs or agents that normalize blood vessels, may enhance the effectiveness of immunotherapy [67, 71].

The application of artificial intelligence (AI) and machine learning (ML) algorithms in cancer vaccine development is growing rapidly. These technologies can analyse vast amounts of data, identify potential antigen targets, and predict the efficacy of vaccine candidates. AI and ML can aid in personalized vaccine design, treatment prediction, and optimization of therapeutic regimens [69].

### 3.3 Personalised Cancer Vaccines

Personalized cancer vaccines, also referred to as individualized or patient-specific cancer vaccines, show great promise in the field of cancer immunotherapy. These vaccines are designed to utilize the body's immune system to identify and target cancer cells with exceptional precision, offering a customized treatment approach for each patient [72].

The core concept underlying personalized cancer vaccines revolves around neoantigens. Neoantigens are distinct proteins or peptides that arise from mutations in a patient's tumour cells. These mutations can lead to the creation of abnormal proteins that are not present in healthy cells. Consequently, neoantigens become attractive targets for the immune system to recognize and attack [73-75].

The development process of personalized cancer vaccines typically involves several stages. It starts with the tumour sequencing which include the acquiring a tumour sample from the patient, which is then subjected to comprehensive sequencing of either the entire exome or genome. This sequencing process aids in identifying the genetic mutations present in the tumour cells, facilitating the detection of neoantigens [76-78]. This is followed by neoantigen prediction where bioinformatics tools are employed to analyse the sequencing data of the tumour and predict the neoantigens that are likely to be immunogenic and capable of triggering an immune response. Various factors are considered during neoantigen prediction, such as their binding affinity to major histocompatibility complex (MHC) molecules, antigen processing and presentation, and recognition by T-cell receptors [79-88]. Neoantigen are selected from the pool of predicted neoantigens and the most suitable candidates are chosen for the formulation of the vaccine. Factors such as predicted immunogenicity, expression levels in the tumour, and the diversity of neoantigens are considered during the selection process [80, 81]. Vaccine are formulated and synthesised once the neoantigens are identified. Different delivery systems can be utilized, including peptides, proteins, viral vectors, or nucleic acids (DNA or RNA). The choice of delivery system depends on factors such as stability, immunogenicity, and ease of manufacturing [83]. Further, the personalized cancer vaccine is administered to the patient, typically through injection. The vaccine's objective is to stimulate the patient's immune system, specifically activating T-cells to recognize and attack tumour cells that bear the neoantigens [83]. Following the administration of the vaccine, the patient's immune response is closely monitored. This process may involve analysing blood samples to evaluate the activation and expansion of neoantigen-specific T-cells. Monitoring the immune response helps determine the effectiveness of the vaccine and guides any necessary adjustments or additional treatment strategies.

Personalized cancer vaccines can be combined with other cancer treatments, such as immune checkpoint inhibitors or traditional therapies like chemotherapy or radiation. The integration of different treatment modalities can enhance the overall anti-tumour response and improve patient outcomes [86, 87, 89]. One significant advantage of personalized cancer vaccines is their capability to target the distinctive characteristics of an individual patient's tumour. By focusing on neoantigens, these vaccines have the potential to generate a more precise and potent immune response compared to non-personalized approaches. However, several challenges need to be addressed for wider adoption of personalized cancer vaccines [84, 85]. These challenges include the complexity and cost of individualized manufacturing processes, the time required for sequencing and vaccine development, and the identification of suitable neoantigens in certain types of tumours [90].

## 4 PROSPECTS OF PRECISION THERAPY IN THE DEVELOPMENT OF CANCER VACCINE

Precision therapy, also referred to as personalized or targeted therapy, has emerged as a ground-breaking approach in the field of cancer treatment. It focuses on identifying and targeting specific genetic and molecular alterations that drive the growth and progression of cancer cells. It employs advanced techniques such as genomic sequencing, proteomic analysis, and molecular profiling to identify key mutations, gene expressions, or abnormal protein signalling pathways unique to an individual's tumour. The insights gained from these analyses facilitate the development of tailored treatments that directly address the specific vulnerabilities of the tumour. In recent years, precision therapy has been integrated into the realm of cancer vaccines, bringing about a revolution in cancer immunotherapy [91, 92]. This integration is well evident through several instances.

### 4.1 Identification of Tumour-specific Antigens

Precision therapy plays a critical role in identifying tumour-specific antigens (TSAs), which are molecules present in cancer cells but not in normal cells. Through the analysis of the tumour's genetic and molecular makeup, researchers can identify unique mutations or aberrations that give rise to TSAs. These TSAs act as targets for the immune system, and when incorporated into cancer vaccines, they trigger a targeted immune response against the cancer cells while sparing healthy tissues [93].

### 4.2 Neoantigens and Personalized Vaccines

Neoantigens are a subtype of TSAs that result from somatic mutations specific to an individual's tumour. Precision therapy aids in identifying these neoantigens by analysing the genomic data of the tumour. Personalized cancer vaccines can then be designed using these neoantigens, ensuring a highly specific immune response against the tumour. The use of neoantigens has shown promising results in clinical trials, demonstrating improved anti-tumour immunity and prolonged patient survival rates [91, 94].

### 4.3 Immune Checkpoint Inhibitors

Precision therapy also plays a crucial role in the development of cancer vaccines that target immune checkpoint inhibitors. Immune checkpoint inhibitors are molecules that regulate the immune response, preventing excessive activation and ensuring immune homeostasis. However, cancer cells can exploit these checkpoint pathways to evade immune surveillance [92]. Precision therapy identifies the specific checkpoint pathways utilized by the tumour cells and enables the development of vaccines that block or modulate these pathways. This approach enhances the immune response against cancer cells, allowing the immune system to recognize and eliminate the tumour more effectively [95].

**Fig. (3):**
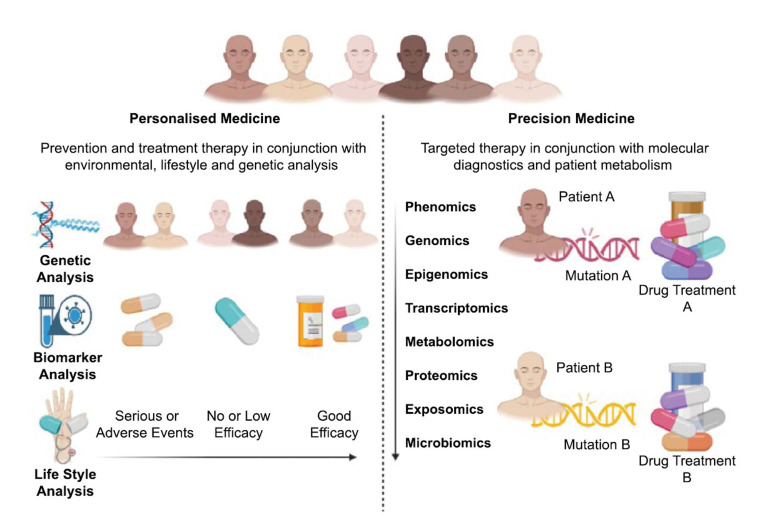
Comparative analysis highlighting the differences between personalised and precision medicine. Personalised approaches work in compliance with traditional physician values while also taking into consideration the patient’s values and coping skills environment. Precision approaches only take personalised methods a notch higher by using genomic data to obtain an efficient drug protocol.

Precision therapy enables the design of individualized cancer vaccines tailored to the specific molecular characteristics of a patient's tumour, maximizing efficacy. By targeting TSAs or immune checkpoint inhibitors, precision therapy precisely directs the immune response toward cancer cells, minimizing collateral damage to healthy tissues [96, 97]. Cancer vaccines developed using precision therapy have the potential to induce long-term immune memory, providing ongoing protection against tumour recurrence (Fig. **3**) [98].

## 5 COMBINATION THERAPY TO ENHANCE THE EFFICIENCY OF CANCER VACCINES

Combination therapy, which involves using multiple treatment modalities concurrently or sequentially, has emerged as a promising approach to enhance the effectiveness of cancer vaccines. By combining various agents such as vaccines, immune checkpoint inhibitors, targeted therapies, and chemotherapy, researchers aim to harness synergistic effects, overcome immune evasion, and improve clinical outcomes for cancer patients [99].

The combination therapy capitalizes on the potential synergistic effects achieved by combining different treatment modalities showing its rationality. By leveraging the distinct mechanisms of action of multiple agents, combination therapy can simultaneously target multiple pathways involved in tumour development, growth, and immune evasion. This comprehensive approach leads to improved clinical responses [98, 100].

Furthermore, such therapy is significant to overcome immune evasion. Cancer cells employ diverse strategies to evade immune recognition and destruction. Combination therapy allows for the targeting of multiple immune checkpoints, including PD-1, CTLA-4, and LAG-3, which are implicated in suppressing antitumour immune responses. By inhibiting these checkpoints while simultaneously stimulating immune activation through vaccines, combination therapy can counteract immune evasion mechanisms and enhance antitumour immunity [101]. Cancer vaccines aim to promote the presentation of tumour-associated antigens to immune cells. However, tumour cells often downregulate antigen-presenting machinery, limiting the effectiveness of vaccines. Combination therapy involving agents that enhance antigen presentation, such as immune modulators or adjuvants, can synergistically enhance vaccine-induced immune responses [100, 102].

### 5.1 Strategies for Combination Therapy

Various strategies are being implemented to enhance the effectiveness of the vaccines *via* combination therapy like simultaneous combination, sequential combination and combinatorial approaches.

The simultaneous combination approach involves administering cancer vaccines and other agents concurrently, capitalizing on their complementary mechanisms of action. For example, combining a cancer vaccine with immune checkpoint inhibitors can boost the primed immune response while preventing immune suppression by inhibitory checkpoints. The administration of cancer vaccines and other agents in a specific order to maximize their effectiveness comes under sequential combination therapy. For instance, priming the immune system with a vaccine to initiate antitumour immunity, followed by the administration of immune checkpoint inhibitors to sustain the activated immune response, has shown promise in preclinical and clinical studies. The combinatorial strategies integrate multiple treatment modalities to exploit synergistic effects. This can include combining cancer vaccines with chemotherapy, targeted therapies, oncolytic viruses, or adoptive cell therapies. These combinations aim to enhance tumour cell killing, modulate the immune response, and overcome resistance mechanisms [102, 103].

**Fig. (4):**
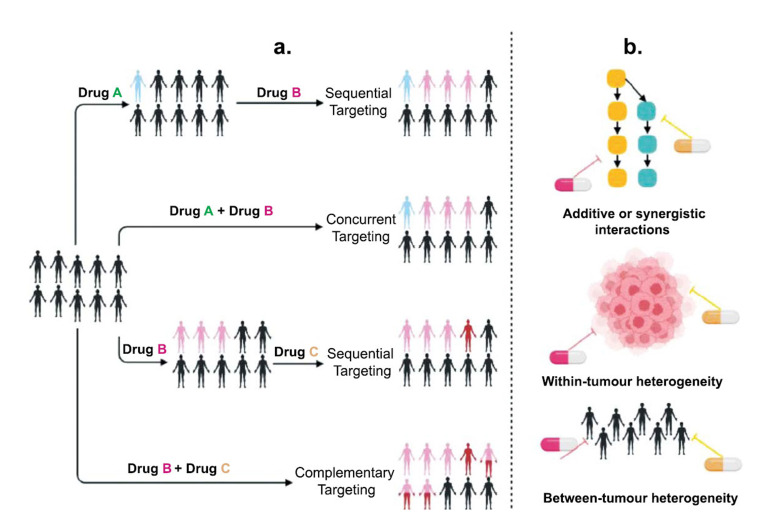
(**a**) Combination therapy aims at several strategies for treatment and therapy. (**b**) Rationale for combination approaches.

Combination therapy has shown promising results in preclinical and clinical studies across various cancer types. Notable successes include the combination of cancer vaccines with immune checkpoint inhibitors in melanoma and lung cancer. However, challenges such as identifying optimal combinations, determining ideal sequencing and dosing, managing toxicities, and understanding resistance mechanisms remain. Future research should focus on refining combination strategies through preclinical models, biomarker discovery, and innovative clinical trial designs to maximize the potential of cancer vaccines in combination therapy (Fig. **4**) [103-105].

## CONCLUSION

In conclusion, cancer vaccines have emerged as a promising field in cancer research and treatment with the potential to revolutionize cancer care. They have the ability to enhance the body's immune system to specifically identify and eliminate cancer cells. Although several cancer vaccines have demonstrated promising results in both preclinical and clinical trials, there is still a need for further refinement to optimize their effectiveness and safety. However, challenges persist in the development of effective cancer vaccines due to the complexity of cancer, its diverse subtypes, and mechanisms of immune evasion. Researchers are focused on designing vaccines that can overcome these challenges and induce robust and long-lasting immune responses. Furthermore, the selection of target antigens, improvement of vaccine delivery methods, and mitigation of potential side effects are areas that require further investigation.

Looking ahead, the future of cancer vaccines appears promising, fuelled by advancements in immunology, genomics, and computational biology. Technologies like next- generation sequencing and high-throughput screening have facilitated more efficient identification of potential vaccine targets. Integration of artificial intelligence and machine learning algorithms also aids in analysing large datasets to identify patterns that can contribute to the development of effective vaccines. Collaboration between academia, industry, and regulatory bodies is crucial for accelerating the development and regulatory approval of cancer vaccines. Streamlining the clinical trial process, improving manufacturing capabilities, and ensuring affordable access to these treatments are also important considerations for the future of cancer vaccines. Continuous research and development, along with collaborative efforts, are key to unlocking the full potential of cancer vaccines and bringing renewed hope to patients worldwide.

## Figures and Tables

**Table 1:** Vaccines for the cancer targeting Tumour-Associated Antigens (TAAs) which have completed trial phase III.

**S. No.**	**Vaccine Name**	**Antigen Name**	**Type of Vaccine**	**Cancer/s**
1.	GI7DT	17 N-terminal amino acids of gastrin	Peptide vaccine	Gastric cancer/Oesophageal cancer
2.	MAGE-A3/NY-ESO-1	Human melanoma antigen A3/ cancer-testis antigen	Peptide vaccine	Multiple myeloma
3.	Nelipepimut-S (NPS)	Human leukocyte antigen (HLA) A2/A3	Peptide vaccine	Breast cancer
4.	gp100	Amino acids 280 through 288 of the melanoma antigen glycoprotein 100	Peptide vaccine	Metastatic melanoma
5.	MDX-1379 (gp100)	Two gp100 melanoma peptides	Peptide vaccine	

**Table 2:** Preventive cancer vaccines approved by the U.S. food and drug administration (FDA).

**S. No.**	**Vaccine Name**	**Vaccine Group**	**Vaccine Composition**	**Target Cancer/s**	**Approval Year**
1.	**Cervarix^®^**	Adjuvanted non-infectious recombinant vaccine	Purified L1 proteins	HPV types 16 and 18 and HPV-related cervical, anal, vulvar, head and neck, penile, and vaginal cancers	2009
2.	**Gardasil^®^**	Non-infectious recombinant vaccine	Purified L1 proteins	HPV types 16, 18, 6 and 11 and HPV-related cervical, anal, vulvar, head and neck, penile, and vaginal cancers	2006
3.	**Gardasil-9^®^**	Non-infectious recombinant vaccine	Purified Virus-Like Particles (VLPs) of major capsid (L1) protein	HPV types 6, 11, 16, 18, 31, 33, 45, 52, and 58 and HPV-related cervical, anal, head and neck, throat, penile, vaginal and vulvar cancers	2014
4.	**Hepatitis B (HBV) vaccine (HEPLISAV-B^®^)**	Recombinant vaccine	Hepatitis B surface antigen	HBV and HBV-related liver cancer	2017

**Table 3:** Therapeutic cancer vaccines approved by the U.S. food and drug administration (FDA).

**S. No.**	**Vaccine Name**	**Receptor**	**Target Cancer/s**	**Approval Year**
1.	**Bacillus Calmette-Guérin (BCG)**	Toll-like receptors (TLR2 and TLR4)	Early-stage bladder cancer	NA
2.	**Sipuleucel-T (Provenge^®^)**	T-cell receptors	Prostate cancer	2010

## LIST OF ABBREVIATIONS

ADCC: Antibody-Dependent-Cellular Cytotoxicity
AFP: Alpha-Fetoprotein
AI: Artificial Intelligence
AMLs: Acute Myeloid Leukaemias
CAR: Chimeric Antigen Receptor
CEA: Carcinoembryonic Antigen
CTAs: Cancer-Testis Antigens
CTLA-4: Cytotoxic T-Lymphocyte-Associated Protein 4
DCs: Dendritic Cells
EBV: Epstein-Barr Virus
EGFRvIII: Epidermal Growth Factor Receptor Variant III
FDA: Food and Drug Administration
GBMs: Glioblastomas
H. pylori: Helicobacter pylori
HBV: Hepatitis Virus Type B
HBV: Hepatitis B Virus
HCC: Hepatocellular Carcinoma
HCV: Hepatitis Virus Type C
HPV: Human Papillomavirus
iCPIs: Immune Check-Point Inhibitors
INDELs: Insertions or Deletions
JNK: c-Jun N-Terminal Kinase Pathway
LAG-3: Lymphocyte-Activation Gene 3
MAGE: Melanoma-Associated Antigen
MAPK: Mitogen-activated Protein Kinase Pathway
MHC: Major Histocompatibility Complex
MHC: Major Histocompatibility Complex
ML: Machine Learning
NPS: Nelipepimut-S
PD-1: Programmed Cell Death Protein 1
PD-L1 siRNA: Programmed Death Ligand 1 Small-Interfering RNA
PSA: Prostate-Specific Antigen
SNVs: Single Nucleotide Variants
TAAs: Tumour-Associated Antigens
TCR: T-Cell Receptor
TILs: Tumour-Infiltrating Lymphocytes
TSAs: Tumour-Specific Antigens
WES: Whole-Exome Sequencing
WGS: Whole-Genome Sequencing
WHO: World Health Organization
WT1: Wilms’ Tumour Protein
